# Pre-operative iron increases haemoglobin concentration before abdominal surgery: a systematic review and meta-analysis of randomized controlled trials

**DOI:** 10.1038/s41598-022-05283-y

**Published:** 2022-02-09

**Authors:** Jeremy Meyer, Roberto Cirocchi, Salomone Di Saverio, Frédéric Ris, James Wheeler, Richard Justin Davies

**Affiliations:** 1 Colorectal Unit, Addenbrooke’s Hospital, Cambridge NHS Foundation Trust, Cambridge, UK; 2 Division of Digestive Surgery, University Hospitals of Geneva, Geneva, Switzerland; 3 Medical School, University of Geneva, Geneva, Switzerland; 4 University of Perugia, Perugia, Italy; 5 Hospital of San Benedetto del Tronto, Marche, Italy

**Keywords:** Medical research, Outcomes research

## Abstract

Professional surgical societies recommend the identification and treatment of pre-operative anaemia in patients scheduled for abdominal surgery. Our aim was to determine if pre-operative iron allows correction of haemoglobin concentration and decreased incidence of peri-operative blood transfusion in patients undergoing major abdominal surgery. MEDLINE, Embase and CENTRAL were searched for RCTs written in English and assessing the effect of pre-operative iron on the incidence of peri-operative allogeneic blood transfusion in patients undergoing major abdominal surgery. Pooled relative risk (RR), risk difference (RD) and mean difference (MD) were obtained using models with random effects. Heterogeneity was assessed using the Q-test and quantified using the I^2^ value. Four RCTs were retained for analysis out of 285 eligible articles. MD in haemoglobin concentration between patients with pre-operative iron and patients without pre-operative iron was of 0.81 g/dl (3 RCTs, 95% CI 0.30 to 1.33, I^2^: 60%, p = 0.002). Pre-operative iron did not lead to reduction in the incidence of peri-operative blood transfusion in terms of RD (4 RCTs, RD: − 0.13, 95% CI − 0.27 to 0.01, I^2^: 65%, p = 0.07) or RR (4 RCTs, RR: 0.57, 95% CI 0.30 to 1.09, I^2^: 64%, p = 0.09). To conclude, pre-operative iron significantly increases haemoglobin concentration by 0.81 g/dl before abdominal surgery but does not reduce the need for peri-operative blood transfusion. Important heterogeneity exists between existing RCTs in terms of populations and interventions. Future trials should target patients suffering from iron-deficiency anaemia and assess the effect of intervention on anaemia-related complications.

## Introduction

The global prevalence of anaemia was estimated to be 32.9% according to data from the Global Burden of Diseases, Injuries and Risk Factors 2010 Study^1^. In patients undergoing colorectal surgery, study of the NSQIP database revealed that 47.4% of patients were suffering from anaemia at the time of surgical admission^2^. The aetiology of anaemia is diverse, and the NHANES III study including 2,814,000 participants showed that about one third of anaemia cases were caused by nutritional deficiencies^3^. In the context of gastrointestinal surgery, this includes anaemia caused by occult bleeding in patients suffering from cancer of the gastrointestinal tract.

Pre-operative anaemia might reflect advanced cancer or poor health status, but can also lead to impaired oxygen delivery to tissues and increased morbidity in the perioperative period. For instance, pre-operative anaemia was reported to be associated with increased post-operative incidence of surgical site infection^4^, increased morbidity^5^, increased mortality^6^ and prolonged length of hospital stay^5^.

Having considered the potential complications caused by pre-operative anaemia, the Enhanced Recovery After Surgery (ERAS) Society recommends to screen for pre-operative anaemia and to correct it when present^7^. Therapeutic interventions include pre- or peri-operative blood transfusion, pre-operative erythropoietin administration^8–11^ and pre-operative iron administration. The National Institute for Health and Care Excellence (NICE) NG24 recommendation advises to restrict erythropoietin administration in the surgical setting for anaemic patients refusing blood transfusion or in case of non-compatibility with available transfusion, and to offer oral iron in patients with iron-deficiency anaemia before surgery^12^.

However, pooled high-quality evidence supporting the use or pre-operative iron in anaemic patients before digestive surgery has been lacking until the recent release of several randomized controlled trials (RCT) in the field^13–16^. Therefore, we aimed to determine if pre-operative iron allows a reduction in the incidence of peri-operative blood transfusion in patients undergoing major abdominal surgery and correcting haemoglobin concentration (Table S1).

## Materials and methods

MEDLINE, Embase and CENTRAL were searched without time limit to 28.12.2020 for RCTs written in English assessing the effect of preoperative iron administration on the incidence of allogeneic blood transfusion (primary outcome) and correction of anaemia (secondary outcome) during and after abdominal surgery (Table S2). References of review articles in the field were screened and considered for inclusion. RCTs comparing preoperative intravenous or oral iron *versus* no iron or placebo in patients undergoing abdominal surgery were retained. Non-randomized studies, letters, secondary analyses of original studies, RCT protocols and trials not reporting the incidence of peri- or post-operative allogeneic blood transfusion in the interventional and control groups were excluded. Two independent reviewers (RC, JM) performed the literature screening. In case of disagreement, consensus was reached with a third author (RJD). Pooled relative risk (RR), risk difference (RD) and mean difference (MD) were obtained using models with random effects. Heterogeneity was assessed using the Q-test and quantified using the I^2^ value. Risk of bias was assessed using the RoB2 Cochrane Collaboration’s tool for assessing risk of bias in RCTs^17^. Publication bias was investigated using funnel plots^18^. The software Review Manager (RevMan 5, version 5.3, Copenhagen: the Nordic Cochrane Centre, The Cochrane Collaboration, 2014) was used for the meta-analysis and the risk of bias assessment. The systematic review and meta-analysis complied with the PRISMA guidelines^19^ (Table S3), respected recommendations in the field^20^ and was registered into the international prospective register of systematic reviews Prospero (CRD42021228806).

## Results

### Selection of articles

**Figure 1:**
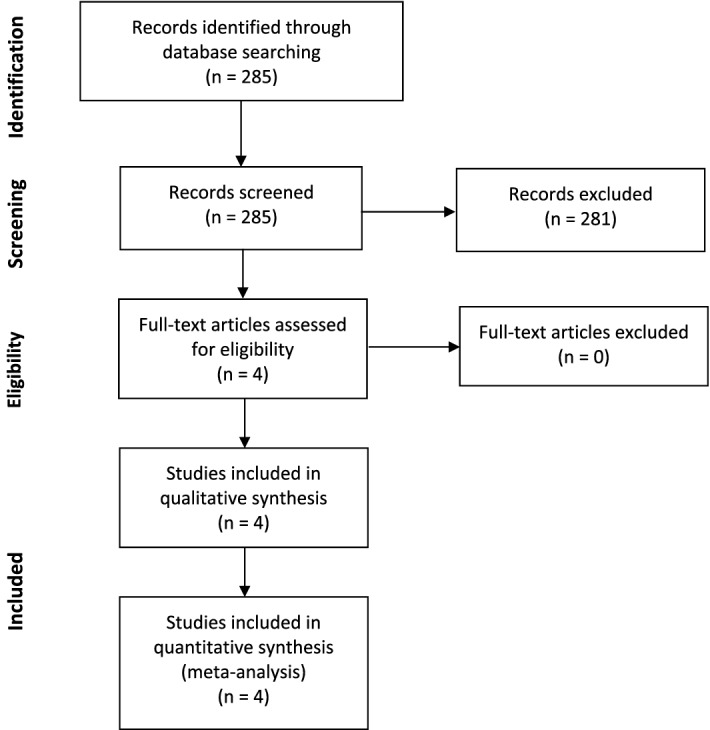
PRISMA flowchart.

Search of databases identified 285 eligible articles. After screening, 281 were excluded for not fulfilling the inclusion criteria or meeting one of the exclusion criteria, and four RCTs^13–16^ were included for analysis (Fig. 1).

### Characteristics of included studies

Two RCTs^13,16^ included patients who underwent major abdominal surgery and two RCTs^14,15^ included only patients who had colorectal surgery. Two trials included only anaemic patients^13,16^, and one of them only patients with iron-deficiency anaemia^13^. Pre-operative iron was given intravenously in 3 RCTs^13,15,16^ and orally in one^14^. Characteristics of included RCTs are summarized in Table 1.

**Table 1:** Characteristics of included studies.

Authors	Year	Country	Acronyme	Period	Patients, n	Population	Intervention	Control	Primary outcome
Richards et al	2020	United Kingdom	PREVENTT	01.2014–09.2018	135	Major open abdominal surgery with anaemia	IV 1000 mg iron 10-42d before surgery	Placebo	Blood transfusion/death from randomization to POD30
Froessler et al	2016	Australia	–	08.2011–11.2014	72	Major open abdominal surgery with iron-deficiency anaemia	IV 15 mg/kg ferric carboxymaltose 4-21d before surgery + 0.5 mg/ml blood loos if ≥ 100 ml before POD2	Usual care	Blood transfusion
Lidder et al	2007	United Kingdom	–	–	45	Colorectal cancer surgery	Oral ferrous sulphate 200 mg 3×/day for 2 weeks before surgery	Usual care	Hemoglobin concentration
Edwards et al	2009	United Kingdom	–	05.2006–08.2008	60	Colorectal cancer surgery	IV 600 mg iron sucrose 14d before surgery	Placebo	Hemoglobin concentration at admission

### Quality assessment of included studies

One RCT^16^ was considered to be of low risk of bias according to the RoB2 Cochrane Collaboration’s tool. Three RCTs^13–15^ were considered to be of high risk of bias. Detailed assessment is reported in Table S4.

### Haemoglobin concentration at admission after pre-operative administration of iron

**Figure 2:**
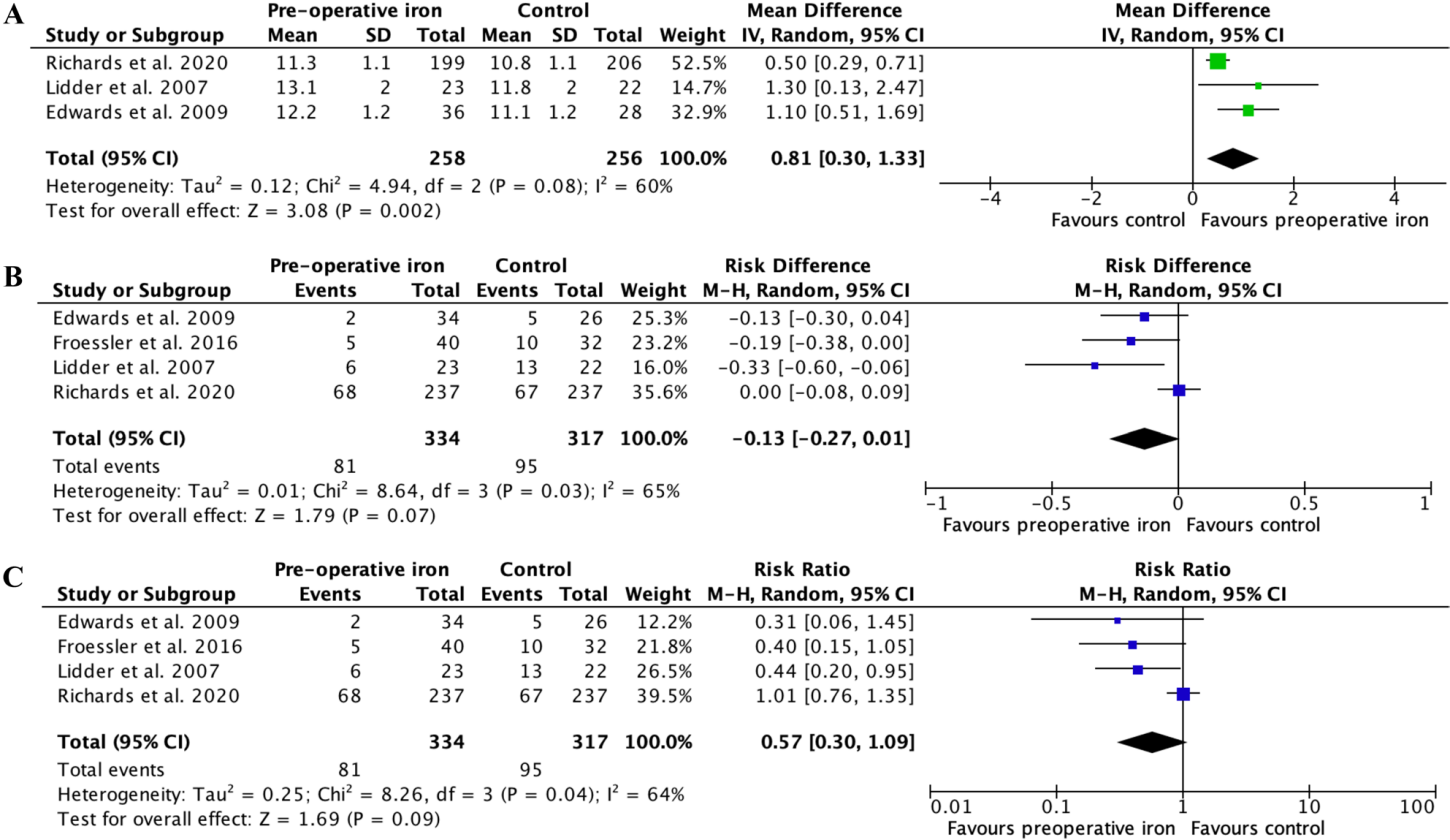
Meta-analysis of the role of pre-operative iron on anaemia outcomes in patients undergoing abdominal surgery. Forest plot comparing pre-operative iron *versus* no pre-operative iron or placebo before abdominal surgery. Each horizontal bar summarizes a study. The bars represent 95% confidence intervals. The grey squares inform on each of the studies’ weight in the meta-analysis. The diamond in the lower part of the graph depicts the pooled estimate along with 95% confidence intervals. Pooled relative risk (RR), risk difference (RD) and mean difference (MD) were obtained using models with random effects. Heterogeneity was assessed using the Q-test and quantified using the I^2^ value. Risk of bias was assessed by using the Cochrane Collaboration’s tool for assessing risk of bias. (**A**) MD in haemoglobin concentration at admission, (**B**) RD for peri-operative allogeneic blood transfusion, (**C**) RR for peri-operative allogeneic blood transfusion. Data for the RCT by Richards et al. were extracted from Table 2 of their article including large blood transfusions for the number of patients who received blood transfusion, and reconstituted from the text (which reported a MD of 4.7 g/l and from Figure 2 for the mean haemoglobin concentration).

**Figure 3:**
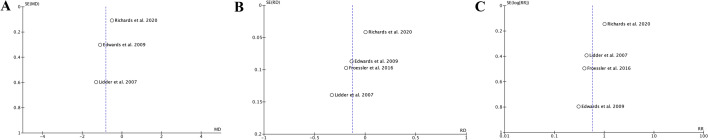
Funnel plots for assessing the risk of publication bias. The standard error is plotted as a function of the observed effect estimate. Vertical bars correspond to pooled estimates from a random effects meta-analysis.

Three RCTs (514 patients)^14–16^ reported the mean haemoglobin concentrations at admission in patients with and without pre-operative iron. The MD in haemoglobin concentration between the two groups of patients was of 0.81 g/dl (95% CI 0.30 to 1.33 g/dl, I^2^: 60%, p = 0.002) in favour of patients who received pre-operative iron (Fig. 2A). This means that patients who benefited from pre-operative iron administration had, on average, a haemoglobin concentration that was higher by 0.81 g/dl (8.1 g/l) than control patients at time of admission. Analysis of symmetry of funnel plot did not identify any potential publication bias (Fig. 3A).

### Risk of perioperative blood transfusion after preoperative administration of iron

The four included RCTs (651 patients)^13–16^ reported the number of patients who received blood transfusion in both the intervention group and the control group. The risk difference in terms of blood transfusion was not significantly reduced by 13 percentage points (RD: − 0.13, 95% CI − 0.27 to 0.01, I^2^: 65%, p = 0.07) in patients who received pre-operative iron when compared to patients who did not receive pre-operative iron (Fig. 2B). The RR to receive blood transfusion was not significantly reduced in patients of the intervention group (RR: 0.57, 95% CI 0.30 to 1.09, I^2^: 64%, p = 0.09) (Fig. 2C). Analysis of the funnel plots for RD and RR (Fig. 3B,C, respectively) was limited by the low number of included RCTs, which did not allow generating 95% intervals. Nevertheless, there was some potential asymmetry caused by the well-powered RCT by Richards et al. Therefore, sensitivity analysis was performed by excluding trials one by one. Excluding the RCT by Richards et al. led the RD to increase to 19 percentage points (95% CI − 0.30 to − 0.07, I^2^: 0%, p = 0.001) in favour of iron, with a pooled result becoming statistically significant and with reduced heterogeneity. Similarly, the RR was of 0.41 (95% CI 0.23 to 0.71, I^2^: 0%, p = 0.002).

## Discussion

The beneficial effect of preoperative iron on haemoglobin concentration and allogeneic blood transfusion was reported by a recent systematic review and meta-analysis pooling data from different surgical specialties^21^. However, no subgroup analysis was performed for patient who underwent abdominal surgery, and the effect on blood transfusion was not reported by another meta-analysis pooling only RCTs^22^. The population of patients undergoing abdominal surgery was specifically explored by a systematic review, which concluded that preoperative iron allowed increasing haemoglobin concentration, but did not allow decreasing the incidence of allogeneic blood transfusion^23^. The FAIRY trial also showed that haemoglobin concentration could also be increased by post-operative administration of iron in anaemic patients after gastrectomy^24^.

In the present systematic review and meta-analysis pooling only RCTs and specifically including patients undergoing abdominal surgery, we showed that administration of pre-operative iron allowed increasing haemoglobin concentration by 0.81 g/dl at time of admission.

However, no significant effect of pre-operative iron was found in terms of perioperative allogeneic blood transfusion, unless the latest RCT by Richards et al.^16^ was excluded from the pooled analysis. In this case, heterogeneity of the results decreased from 64 to 0%. However, the trial by Richards et al. is also the one with the biggest sample size and no reason exists for excluding it from the pooled analysis.

By looking closely at the PICO questions of existing RCTs in the field, as summarized in Table S3, we noted that three^14–16^ out of the four included RCT, including the RCT by Richard et al., did not specifically include patients suffering from iron-deficiency anaemia, therefore preventing us from performing the planned sensitivity analysis based on the presence or absence of iron-deficiency anaemia. As we have previously commented in relation to the PREVENTT trial^25^, this might lead to statistical underpowering of these trials in the evaluation of the effect of pre-operative iron. In addition, Lidder et al. included a total of only six anaemic patients in the intervention group and 14 in the control group. Considering that iron-deficiency anaemia represents about one third of anaemia causes^3^, the iron intervention might only have an effect on two patients in the intervention group, which is far too small to show any potential effect of pre-operative iron even in case of an extremely efficient treatment^14^. Moreover, five patients belonging to the control group received IV iron.

Included trials were also underpowered when comparing the total numbers of patients included with the numbers of patients initially planned. For instance, Edwards et al. included 60 patients in their final analysis and showed no significant effect of pre-operative iron, but their initial study protocol registered into the EU clinical trials register (2005-003608-13) indicated 126 patients^15^. Froessler et al. reported a sample size calculation including 134 patients per group^13^. However, in the published article, only 72 patients were included: 40 in the intervention group and 32 in the control group. Nevertheless, it appears that the trial was terminated earlier than expected due to poorer outcome in the control group. Therefore, when targeting only patients with iron-deficiency anaemia, an effect of pre-operative iron on the incidence of peri-operative transfusion can be noticeable even with low numbers of patients.

Considering the limitations of existing RCTs, we recommend that future trials only include anaemic patients with iron-deficiency or, in case of a pragmatic approach including all anaemic patients, to at least perform the sample size calculation based on patients with iron-deficiency anaemia. This would be in line with the NICE guideline^12^, which recommend administering pre-operative iron specifically in patients suffering from iron-deficiency anaemia, and would be preferable in terms of patient safety (patients without iron-deficiency anaemia should not receive intravenous iron) and health economics.

Another potential source of heterogeneity was the timing and mode of administration of pre-operative iron, which raised some concerns regarding efficiency of trials interventions. For instance, in included RCTs, iron was given 2 weeks^14,15^, 4–21 days^13^ or 10–42 days^16^ before surgery, although haemoglobin concentration was shown to increase over time after administration of iron^26^. Moreover, we note that a recent prospective observational study including 1′728 surgical patients showed that iron supplementation allowed to decrease the incidence of post-operative blood transfusion in iron-deficient patients only if it was given more than 7 days before the surgery^27^, which corresponds to the time required for erythropoiesis. In addition, 3 RCTs administered intravenous iron^13,15,16^ and one provided oral iron^14^. Although intravenous iron was shown to be more effective in correcting iron-deficiency anaemia in abdominal surgery, no difference could be found between intravenous and oral iron in terms of peri-operative blood transfusion, notably by the IVICA trial^28,29^.

We also noted that included RCT reported the incidence of peri-operative blood transfusion as the main or secondary outcome to measure the efficiency of pre-operative iron on the prevention of anaemia-related complications. We believe that the indication for peri-operative blood transfusion based on haemoglobin concentration is subject to heterogeneity^30^ unless explicitly specified in the trial protocol. None of the included trials used strict criteria for blood transfusion, which was most often the result of decision of the anaesthetic team^14,15^. Further, peri-operative blood transfusion is the treatment of anaemia and not a consequence of poor tissue perfusion and hypoxia. Peri-operative blood transfusion might therefore constitute a confounding factor of the effects of anaemia, and measuring directly the effects of anaemia to assess the efficiency of the trial intervention (iron) might potentially show an effect of that intervention.

Finally, we note that 3 out of the 4 RCTs included in the meta-analysis were considered to be of high risk of bias, as assessed by the RoB2 Cochrane Collaboration tool.

Therefore, we think that future RCTs in the field should also report the incidence of anaemia-related complications in the intervention and in the control groups. In abdominal surgery, this should include reporting the incidence of surgical site infection and anastomotic leak. In this regards, it is noteworthy to mention that the long-term follow-up of the IVICA trial reported that patients with colorectal cancer who responded to correction of pre-operative anaemia had improved 5-year overall survival compared with patients who did not respond to iron^31^, therefore questioning about the choice of outcomes to assess the potential beneficial effect of administration of iron. An analysis of the findings of our meta-analysis (pre-operative iron allows to increase haemoglobin concentration at time of admission) in the light of other outcomes than peri-operative blood transfusion would be of interest.

In conclusion, pre-operative iron significantly increases haemoglobin concentration by 0.81 g/dl (8.1 g/l) before abdominal surgery but does not reduce the need for peri-operative blood transfusion. Important heterogeneity exists between available RCTs in terms of populations and interventions. Future trials in the field^32^ should target patients suffering from iron-deficiency anaemia and assess the effect of intervention on anaemia-related complications.

## Supplementary Information

Supplementary Table S1.

Supplementary Table S2.

Supplementary Table S3.

Supplementary Table S4.
