# Author Correction: Pre-operative iron increases haemoglobin concentration before abdominal surgery: a systematic review and meta-analysis of randomized controlled trials

**DOI:** 10.1038/s41598-022-10305-w

**Published:** 2022-04-12

**Authors:** Jeremy Meyer, Roberto Cirocchi, Salomone Di Saverio, Frédéric Ris, James Wheeler, Richard Justin Davies

**Affiliations:** 1 Colorectal Unit, Addenbrooke’s Hospital, Cambridge NHS Foundation Trust, Cambridge, UK; 2 Division of Digestive Surgery, University Hospitals of Geneva, Geneva, Switzerland; 3 Medical School, University of Geneva, Geneva, Switzerland; 4 University of Perugia, Perugia, Italy; 5 Hospital of San Benedetto del Tronto, Marche, Italy

Correction to: *Scientific Reports* 10.1038/s41598-022-05283-y, published online 09 February 2022

The original version of this Article contained an error in the Discussion section,

“As we have previously commented in relation to the PREVENTT trial^25^, this might lead to statistical underpowering of these trials in the evaluation of the effect of pre-operative iron. Of note, in the trial by Richards et al.^16^, proportions of iron deficient-patients were of 28% and 29%, depending on the group.”

now reads:

“As we have previously commented in relation to the PREVENTT trial^25^, this might lead to statistical underpowering of these trials in the evaluation of the effect of pre-operative iron.”

The original Article has been corrected.

