# Supplementary material for: Pre-operative iron increases haemoglobin concentration before abdominal surgery: a systematic review and meta-analysis of randomized controlled trials
Source: Sci Rep. 2022 Feb 9;12:2158. doi: 10.1038/s41598-022-05283-y (PMC8828750; doi:10.1038/s41598-022-05283-y)
Supplement: Supplementary file 1 — Supplementary Table S1. [file 41598_2022_5283_MOESM1_ESM.docx]

**Table S1. PICOS table**

| Population | Patients undergoing abdominal surgery |
| --- | --- |
| Intervention | Pre-operative administration of iron |
| Control | No administration of iron |
| Outcome | Primary : incidence of peri-operative blood transfusion, secondary: Hb concentration at admission for surgery |
| Study design | Randomized controlled trials |
