# Supplementary material for: Pre-operative iron increases haemoglobin concentration before abdominal surgery: a systematic review and meta-analysis of randomized controlled trials
Source: Sci Rep. 2022 Feb 9;12:2158. doi: 10.1038/s41598-022-05283-y (PMC8828750; doi:10.1038/s41598-022-05283-y)
Supplement: Supplementary file 2 — Supplementary Table S2. [file 41598_2022_5283_MOESM2_ESM.docx]

| **Database** | **Search build** | **Occurrences** |
| --- | --- | --- |
| MEDLINE | (iron[Title/Abstract]) AND ((surg*[Title/Abstract]) OR (op*[Title/Abstract])) AND ((RCT[Title/Abstract]) OR (randomized controlled trial[Title/Abstract]) OR (randomised controlled trial[Title/Abstract])) | 55 |
| EMBASE | 'iron':ti,ab,kw AND 'abdominal surgery':ti,ab,kw AND 'randomized controlled trial':ti,ab,kw | 7 |
| COCHRANE CENTRAL | "iron" in Title Abstract Keyword AND "surgery" in Title Abstract Keyword AND "randomized controlled trial" in Publication Type - (with Word variations) | 222 |
| Other sources | - | 1 |

**Table S2. Literature search strategy**
