# Supplementary material for: Pre-operative iron increases haemoglobin concentration before abdominal surgery: a systematic review and meta-analysis of randomized controlled trials
Source: Sci Rep. 2022 Feb 9;12:2158. doi: 10.1038/s41598-022-05283-y (PMC8828750; doi:10.1038/s41598-022-05283-y)
Supplement: Supplementary file 4 — Supplementary Table S4. [file 41598_2022_5283_MOESM4_ESM.docx]

| **Study details**   \| **Reference** \| Randomized clinical trial of preoperative intravenous iron sucrose to reduce blood transfusion in anaemic patients after  colorectal cancer surgery  *T. J. Edwards, E. J. Noble, A. Durran, N. Mellor and K. B. Hosie* \| \| --- \| --- \|   **Study design**   \| X \| Individually-randomized parallel-group trial \| \| --- \| --- \| \| □ \| Cluster-randomized parallel-group trial \| \| □ \| Individually randomized cross-over (or other matched) trial \|   **For the purposes of this assessment, the interventions being compared are defined as**   \| Experimental: \| Two infusions of 250ml intravenous placebo (0.9 % saline) \| Comparator: \| Two infusions of 300 mg iron sucrose made up to 250 ml with 0.9% saline, provided as 15ml ferric hydroxide with sucrose in a 2 per cent solution (Venofer; Syner-Med, Purley, UK). \| \| --- \| --- \| --- \| --- \|  \| **Specify which outcome is being assessed for risk of bias** \| Incidence of perioperative blood transfusion. \| \| --- \| --- \|  \| **Specify the numerical result being assessed.** In case of multiple alternative analyses being presented, specify the numeric result (e.g. RR = 1.52 (95% CI 0.83 to 2.77) and/or a reference (e.g. to a table, figure or paragraph) that uniquely defines the result being assessed. \| Proportion of patients who received perioperative blood transfusion. \| \| --- \| --- \|   **Is the review team’s aim for this result…?**   \| □ \| to assess the effect of *assignment to intervention* (the ‘intention-to-treat’ effect) \| \| --- \| --- \| \| X \| to assess the effect of *adhering to intervention* (the ‘per-protocol’ effect) \|   **If the aim is to assess the effect of *adhering to intervention***, select the deviations from intended intervention that should be addressed (at least one must be checked):  □ occurrence of non-protocol interventions  X failures in implementing the intervention that could have affected the outcome  □ non-adherence to their assigned intervention by trial participants  **Which of the following sources were obtained to help inform the risk-of-bias assessment? (tick as many as apply)**  X Journal article(s) with results of the trial  □ Trial protocol  □ Statistical analysis plan (SAP)  □ Non-commercial trial registry record (e.g. ClinicalTrials.gov record)  □ Company-owned trial registry record (e.g. GSK Clinical Study Register record)  □ “Grey literature” (e.g. unpublished thesis)  □ Conference abstract(s) about the trial  □ Regulatory document (e.g. Clinical Study Report, Drug Approval Package)  □ Research ethics application  □ Grant database summary (e.g. NIH RePORTER or Research Councils UK Gateway to Research)  □ Personal communication with trialist  □ Personal communication with the sponsor |
| --- | --- | --- | --- | --- | --- | --- | --- | --- | --- | --- | --- | --- | --- | --- | --- | --- | --- | --- | --- | --- |

## Risk of bias assessment

Responses underlined in green are potential markers for low risk of bias, and responses in red are potential markers for a risk of bias. Where questions relate only to sign posts to other questions, no formatting is used.

**Domain 1: Risk of bias arising from the randomization process**

| **Signalling questions** | **Comments** | **Response options** |
| --- | --- | --- |
| **1.1 Was the allocation sequence random?** | Yes, “Computer-generated randomization sequence provided by the Research and Development Support Unit » | Y |
| **1.2 Was the allocation sequence concealed until participants were enrolled and assigned to interventions?** | Yes, « Allocation codes were sealed in sequentially numbered opaque envelopes which were secured within a locked store room in a dedicated research unit (this was remote from the clinical areas of the hospital where participants were to undergo outpatient, ward and operative treatment). Only after recruitment was an envelope opened by the investigator administering the infusion, following the inscribed strict numerical order and for the relevant subset appropriate to the Hb status of the participant.” | Y |
| **1.3 Did baseline differences between intervention groups suggest a problem with the randomization process?** | Table 1, but no p-values were reported. | PN |
| **Risk-of-bias judgement** | **Low risk of bias.** | **Low** |

Domain 2: Risk of bias due to deviations from the intended interventions (*effect of adhering to intervention*)

| **Signalling questions** | **Comments** | **Response options** |
| --- | --- | --- |
| **2.1. Were participants aware of their assigned intervention during the trial?** | “Although the investigator administering the infusion was not blinded to the treatment group, this was concealed from the patient by using an opaque sheath to cover the drug giving set.” | N |
| **2.2. Were carers and people delivering the interventions aware of participants' assigned intervention during the trial?** | “Although the investigator administering the infusion was not blinded to the treatment group, this was concealed from the patient by using an opaque sheath to cover the drug giving set.”  “The chief investigator and clinicians involved in perioperative care also remained blinded to the treatment group for the duration of the trial.” | PY |
| **2.3. [If applicable:] If Y/PY/NI to 2.1 or 2.2: Were important non-protocol interventions balanced across intervention groups?** | No information | NI |
| **2.4. [If applicable:] Were there failures in implementing the intervention that could have affected the outcome?** | One patient in each group did not attend the second infusion. However, the analysis was performed per protocol (despite being indicated as intention-to-treat). In addition, “in two patients surgery had to be performed before  the prescribed 14 days owing to constraints of the cancer  waiting targets) », but we don’t know the allocation group of these patients. | PY |
| **2.5. [If applicable:] Was there non-adherence to the assigned intervention regimen that could have affected participants’ outcomes?** | One patient in each group did not attend the second infusion. However, the analysis was performed per protocol (despite being indicated as intention-to-treat). | PN |
| **2.6. If N/PN/NI to 2.3, or Y/PY/NI to 2.4 or 2.5: Was an appropriate analysis used to estimate the effect of adhering to the intervention?** | Per protocol analysis, | N |
| **Risk-of-bias judgement** | **High risk of bias.** | **High** |

Domain 3: Missing outcome data

| **Signalling questions** | **Comments** | **Response options** |
| --- | --- | --- |
| **3.1 Were data for this outcome available for all, or nearly all, participants randomized?** | No information was provided regarding outcome data for perioperative blood transfusion. | NI |
| **3.2 If N/PN/NI to 3.1: Is there evidence that the result was not biased by missing outcome data?** | No information was provided regarding outcome data for perioperative blood transfusion. | NI |
| **3.3 If N/PN to 3.2: Could missingness in the outcome depend on its true value?** | No information was provided regarding outcome data for perioperative blood transfusion. | NI |
| **3.4 If Y/PY/NI to 3.3: Is it likely that missingness in the outcome depended on its true value?** | No information was provided regarding outcome data for perioperative blood transfusion. | NI |
| **Risk-of-bias judgement** | **High risk of bias** | **High** |

Domain 4: Risk of bias in measurement of the outcome

| **Signalling questions** | **Comments** | **Response options** |
| --- | --- | --- |
| **4.1 Was the method of measuring the outcome inappropriate?** | No. The assessed outcome is perioperative blood transfusion. | N |
| **4.2 Could measurement or ascertainment of the outcome have differed between intervention groups?** | No | N |
| **4.3 If N/PN/NI to 4.1 and 4.2: Were outcome assessors aware of the intervention received by study participants?** | “The chief investigator and clinicians involved in perioperative care also remained blinded to the treatment group for the duration of the trial.” | N |
| **4.4 If Y/PY/NI to 4.3: Could assessment of the outcome have been influenced by knowledge of intervention received?** | N/a | NA |
| **4.5 If Y/PY/NI to 4.4:** **Is it likely that assessment of the outcome was influenced by knowledge of intervention received?** | N/a | NA |
| **Risk-of-bias judgement** | **Low risk of bias** | **Low** |

Domain 5: Risk of bias in selection of the reported result

| **Signalling questions** | **Comments** | **Response options** |
| --- | --- | --- |
| **5.1 Were the data that produced this result analysed in accordance with a pre-specified analysis plan that was finalized before unblinded outcome data were available for analysis?** | The trial was sponsored by the in-hospital research and development  unit and registered with the UK Medicines and Healthcare products Regulatory Agency (registration number: 2005- 003 608-13). No mention of registration into clinicaltrials.gov is reported. | PY |
| **Is the numerical result being assessed likely to have been selected, on the basis of the results, from...** |  |  |
| **5.2. ... multiple eligible outcome measurements (e.g. scales, definitions, time points) within the outcome domain?** | No | N |
| **5.3 ... multiple eligible analyses of the data?** | No | N |
| **Risk-of-bias judgement** | **Low risk of bias** | **Low** |

Overall risk of bias

| **Risk-of-bias judgement** | **High risk of bias** | **High** |
| --- | --- | --- |

| **Study details**   \| **Reference** \| The Important Role for Intravenous Iron in Perioperative Patient Blood Management in Major Abdominal Surgery  *B. Froessler, P. Palm, I. Weber, N. Hodyl, R. Singh, E. Murphy* \| \| --- \| --- \|   **Study design**   \| X \| Individually-randomized parallel-group trial \| \| --- \| --- \| \| □ \| Cluster-randomized parallel-group trial \| \| □ \| Individually randomized cross-over (or other matched) trial \|   **For the purposes of this assessment, the interventions being compared are defined as**   \| Experimental: \| 4-21 days before surgery: IV ferric carboxymaltose 15 mg/kg bodyweight to a maximum  dose of 1000 mg given 4-12 days before surgery, plus  0.5mg of ferric carboxymaltose  per recorded 1mL of blood loss, if blood loss was at least 100mL, within 2 days after surgery. \| Comparator: \| Anemia management, provided by the primary care physician or  surgical home team, consisting in either no treatment,  continued observations, oral iron recommendations or blood transfusion. Prescription and administration of IV iron was not disallowed. \| \| --- \| --- \| --- \| --- \|  \| **Specify which outcome is being assessed for risk of bias** \| Incidence of blood transfusion. \| \| --- \| --- \|  \| **Specify the numerical result being assessed.** In case of multiple alternative analyses being presented, specify the numeric result (e.g. RR = 1.52 (95% CI 0.83 to 2.77) and/or a reference (e.g. to a table, figure or paragraph) that uniquely defines the result being assessed. \| Proportion of patients who received perioperative blood transfusion. \| \| --- \| --- \|   **Is the review team’s aim for this result…?**   \| X \| to assess the effect of *assignment to intervention* (the ‘intention-to-treat’ effect) \| \| --- \| --- \| \| X \| to assess the effect of *adhering to intervention* (the ‘per-protocol’ effect) \|   **If the aim is to assess the effect of *adhering to intervention***, select the deviations from intended intervention that should be addressed (at least one must be checked):  □ occurrence of non-protocol interventions  □ failures in implementing the intervention that could have affected the outcome  □ non-adherence to their assigned intervention by trial participants  **Which of the following sources were obtained to help inform the risk-of-bias assessment? (tick as many as apply)**  X Journal article(s) with results of the trial  □ Trial protocol  □ Statistical analysis plan (SAP)  □ Non-commercial trial registry record (e.g. ClinicalTrials.gov record)  □ Company-owned trial registry record (e.g. GSK Clinical Study Register record)  □ “Grey literature” (e.g. unpublished thesis)  □ Conference abstract(s) about the trial  □ Regulatory document (e.g. Clinical Study Report, Drug Approval Package)  □ Research ethics application  □ Grant database summary (e.g. NIH RePORTER or Research Councils UK Gateway to Research)  □ Personal communication with trialist  □ Personal communication with the sponsor |
| --- | --- | --- | --- | --- | --- | --- | --- | --- | --- | --- | --- | --- | --- | --- | --- | --- | --- | --- | --- | --- |

## Risk of bias assessment

Responses underlined in green are potential markers for low risk of bias, and responses in red are potential markers for a risk of bias. Where questions relate only to sign posts to other questions, no formatting is used.

**Domain 1: Risk of bias arising from the randomization process**

| **Signalling questions** | **Comments** | **Response options** |
| --- | --- | --- |
| **1.1 Was the allocation sequence random?** | « Randomization followed a computer-generated number sequence » | Y |
| **1.2 Was the allocation sequence concealed until participants were enrolled and assigned to interventions?** | “Allocation was conducted by telephone” | Y |
| **1.3 Did baseline differences between intervention groups suggest a problem with the randomization process?** | Table 1, but no p-values were reported. Also, there were 40 patients randomized to the intervention group, and only 32 to the control group. | PY |
| **Risk-of-bias judgement** | **Some concerns** | **Some concerns** |

Domain 2: Risk of bias due to deviations from the intended interventions (*effect of assignment to intervention*)

| **Signalling questions** | **Comments** | **Response options** |
| --- | --- | --- |
| **2.1. Were participants aware of their assigned intervention during the trial?** | Control patients did not receive placebo, so yes. | Y |
| **2.2. Were carers and people delivering the interventions aware of participants' assigned intervention during the trial?** | « The surgeon performing the operation was informed of patient participation in the study but group allocation was not revealed.”  However, the investigator delivering the iron infusion was probably aware of the assigned intervention. | PY |
| **2.3. If Y/PY/NI to 2.1 or 2.2: Were there deviations from the intended intervention that arose because of the trial context?** | “Five participants in the usual care group were given a median IV iron dose of 1800mg (IQR 1467–2000).” | Y |
| **2.4 If Y/PY to 2.3: Were these deviations likely to have affected the outcome?** | Yes, if we consider that iron has an effect. | Y |
| **2.5. If Y/PY/NI to 2.4: Were these deviations from intended intervention balanced between groups?** | No, it only concerned the control group. | N |
| **2.6 Was an appropriate analysis used to estimate the effect of assignment to intervention?** | Yes | Y |
| **2.7 If N/PN/NI to 2.6: Was there potential for a substantial impact (on the result) of the failure to analyse participants in the group to which they were randomized?** | N/a | NA |
| **Risk-of-bias judgement** | **High risk of bias** | **High** |

Domain 3: Missing outcome data

| **Signalling questions** | **Comments** | **Response options** |
| --- | --- | --- |
| **3.1 Were data for this outcome available for all, or nearly all, participants randomized?** | No information was provided regarding outcome data for perioperative blood transfusion. | NI |
| **3.2 If N/PN/NI to 3.1: Is there evidence that the result was not biased by missing outcome data?** | No information was provided regarding outcome data for perioperative blood transfusion. | NI |
| **3.3 If N/PN to 3.2: Could missingness in the outcome depend on its true value?** | No information was provided regarding outcome data for perioperative blood transfusion. | NI |
| **3.4 If Y/PY/NI to 3.3: Is it likely that missingness in the outcome depended on its true value?** | No information was provided regarding outcome data for perioperative blood transfusion. | NI |
| **Risk-of-bias judgement** | **High risk of bias** | **High** |

Domain 4: Risk of bias in measurement of the outcome

| **Signalling questions** | **Comments** | **Response options** |
| --- | --- | --- |
| **4.1 Was the method of measuring the outcome inappropriate?** | No. The assessed outcome is perioperative blood transfusion. | N |
| **4.2 Could measurement or ascertainment of the outcome have differed between intervention groups?** | No | N |
| **4.3 If N/PN/NI to 4.1 and 4.2: Were outcome assessors aware of the intervention received by study participants?** | No information | NI |
| **4.4 If Y/PY/NI to 4.3: Could assessment of the outcome have been influenced by knowledge of intervention received?** | Perioperative blood transfusion is usually indicated into the patient’s chart by the team in charge of the patient. | N |
| **4.5 If Y/PY/NI to 4.4: Is it likely that assessment of the outcome was influenced by knowledge of intervention received?** | N/a | N/a |
| **Risk-of-bias judgement** | **Some concerns** | **Some concerns** |

Domain 5: Risk of bias in selection of the reported result

| **Signalling questions** | **Comments** | **Response options** |
| --- | --- | --- |
| **5.1 Were the data that produced this result analysed in accordance with a pre-specified analysis plan that was finalized before unblinded outcome data were available for analysis?** | “The protocol was approved by the study hospital’s human research ethics committee and registered with the Australian New Zealand Clinical Trials  Registry (ACTRN12611000387921). “No mention of registration into clinicaltrials.gov is reported. | PY |
| **Is the numerical result being assessed likely to have been selected, on the basis of the results, from...** |  |  |
| **5.2. ... multiple eligible outcome measurements (e.g. scales, definitions, time points) within the outcome domain?** | No | N |
| **5.3 ... multiple eligible analyses of the data?** | No | N |
| **Risk-of-bias judgement** | **Low risk of bias** | **Low** |

Overall risk of bias

| **Risk-of-bias judgement** | **High risk of bias** | **High** |
| --- | --- | --- |

| **Study details**   \| **Reference** \| Pre-operative oral iron supplementation reduces blood transfusion in colorectal surgery – a prospective, randomised,controlled trial  *P. G. Lidder, G. Sanders, E. Whitehead, W. J. Douie, N. Mellor, S. J. Lewis, K. B. Hosie* \| \| --- \| --- \|   **Study design**   \| X \| Individually-randomized parallel-group trial \| \| --- \| --- \| \| □ \| Cluster-randomized parallel-group trial \| \| □ \| Individually randomized cross-over (or other matched) trial \|   **For the purposes of this assessment, the interventions being compared are defined as**   \| Experimental: \| Ferrous sulphate 200 mg TDS until surgery \| Comparator: \| Standard clinical management \| \| --- \| --- \| --- \| --- \|  \| **Specify which outcome is being assessed for risk of bias** \| Incidence of blood transfusion. \| \| --- \| --- \|  \| **Specify the numerical result being assessed.** In case of multiple alternative analyses being presented, specify the numeric result (e.g. RR = 1.52 (95% CI 0.83 to 2.77) and/or a reference (e.g. to a table, figure or paragraph) that uniquely defines the result being assessed. \| Proportion of patients who received perioperative blood transfusion. \| \| --- \| --- \|   **Is the review team’s aim for this result…?**   \| X \| to assess the effect of *assignment to intervention* (the ‘intention-to-treat’ effect) \| \| --- \| --- \| \|  \| to assess the effect of *adhering to intervention* (the ‘per-protocol’ effect) \|   **If the aim is to assess the effect of *adhering to intervention***, select the deviations from intended intervention that should be addressed (at least one must be checked):  □ occurrence of non-protocol interventions  □ failures in implementing the intervention that could have affected the outcome  □ non-adherence to their assigned intervention by trial participants  **Which of the following sources were obtained to help inform the risk-of-bias assessment? (tick as many as apply)**  X Journal article(s) with results of the trial  □ Trial protocol  □ Statistical analysis plan (SAP)  □ Non-commercial trial registry record (e.g. ClinicalTrials.gov record)  □ Company-owned trial registry record (e.g. GSK Clinical Study Register record)  □ “Grey literature” (e.g. unpublished thesis)  □ Conference abstract(s) about the trial  □ Regulatory document (e.g. Clinical Study Report, Drug Approval Package)  □ Research ethics application  □ Grant database summary (e.g. NIH RePORTER or Research Councils UK Gateway to Research)  □ Personal communication with trialist  □ Personal communication with the sponsor |
| --- | --- | --- | --- | --- | --- | --- | --- | --- | --- | --- | --- | --- | --- | --- | --- | --- | --- | --- | --- | --- |

## Risk of bias assessment

Responses underlined in green are potential markers for low risk of bias, and responses in red are potential markers for a risk of bias. Where questions relate only to sign posts to other questions, no formatting is used.

**Domain 1: Risk of bias arising from the randomization process**

| **Signalling questions** | **Comments** | **Response options** |
| --- | --- | --- |
| **1.1 Was the allocation sequence random?** | “patients were randomised (by telephone to a distant centre)” | NI |
| **1.2 Was the allocation sequence concealed until participants were enrolled and assigned to interventions?** | “patients were randomised (by telephone to a distant centre)” | Y |
| **1.3 Did baseline differences between intervention groups suggest a problem with the randomization process?** | « There was no significant difference between the two groups in terms of age, sex, operative procedure, operative duration, estimated blood loss, or tumour stage (Tables 2 and 3). » | N |
| **Risk-of-bias judgement** | **Low risk of bias** | **Low** |

Domain 2: Risk of bias due to deviations from the intended interventions (*effect of assignment to intervention*)

| **Signalling questions** | **Comments** | **Response options** |
| --- | --- | --- |
| **2.1. Were participants aware of their assigned intervention during the trial?** | “It was not possible to use a placebo and blind the patient, as  oral iron alters stool colour.” | Y |
| **2.2. Were carers and people delivering the interventions aware of participants' assigned intervention during the trial?** | “The clinical team (surgeons, nurses, anaesthetists) were blinded to treatment allocation.”  However, the carer delivering the intervention (iron) was probably aware of the assigned intervention. | PN |
| **2.3. If Y/PY/NI to 2.1 or 2.2: Were there deviations from the intended intervention that arose because of the trial context?** | Not reported. | N |
| **2.4 If Y/PY to 2.3: Were these deviations likely to have affected the outcome?** | N/a | N/a |
| **2.5. If Y/PY/NI to 2.4: Were these deviations from intended intervention balanced between groups?** | N/a | N/a |
| **2.6 Was an appropriate analysis used to estimate the effect of assignment to intervention?** | « All analysis was performed on an intention-to-treat basis with P < 0.05  taken as being significant », but no additional details were provided. | PY |
| **2.7 If N/PN/NI to 2.6: Was there potential for a substantial impact (on the result) of the failure to analyse participants in the group to which they were randomized?** | N/a | NA |
| **Risk-of-bias judgement** | **Low risk of bias** | **Low** |

Domain 3: Missing outcome data

| **Signalling questions** | **Comments** | **Response options** |
| --- | --- | --- |
| **3.1 Were data for this outcome available for all, or nearly all, participants randomized?** | No information was provided regarding potential missing outcome data for perioperative blood transfusion. | NI |
| **3.2 If N/PN/NI to 3.1: Is there evidence that the result was not biased by missing outcome data?** | No information was provided regarding potential missing outcome data for perioperative blood transfusion. | NI |
| **3.3 If N/PN to 3.2: Could missingness in the outcome depend on its true value?** | No information was provided regarding potential missing outcome data for perioperative blood transfusion. | NI |
| **3.4 If Y/PY/NI to 3.3: Is it likely that missingness in the outcome depended on its true value?** | No information was provided regarding potential missing outcome data for perioperative blood transfusion. | NI |
| **Risk-of-bias judgement** | **High risk of bias** | **High** |

Domain 4: Risk of bias in measurement of the outcome

| **Signalling questions** | **Comments** | **Response options** |
| --- | --- | --- |
| **4.1 Was the method of measuring the outcome inappropriate?** | No. The assessed outcome is perioperative blood transfusion. | N |
| **4.2 Could measurement or ascertainment of the outcome have differed between intervention groups?** | No | N |
| **4.3 If N/PN/NI to 4.1 and 4.2: Were outcome assessors aware of the intervention received by study participants?** | “The collection of data was performed by a research fellow not involved in the direct care of the patient, and gathered from the clinical notes.” Occurrence of blood transfusion was probably recorded by the anesthetic team.  “The clinical team (surgeons, nurses, anaesthetists) were blinded to treatment allocation.” | N |
| **4.4 If Y/PY/NI to 4.3: Could assessment of the outcome have been influenced by knowledge of intervention received?** | Perioperative blood transfusion is usually indicated into the patient’s chart by the team in charge of the patient. | N |
| **4.5 If Y/PY/NI to 4.4: Is it likely that assessment of the outcome was influenced by knowledge of intervention received?** | N/a | N/a |
| **Risk-of-bias judgement** | **Low risk of bias** | **Low** |

Domain 5: Risk of bias in selection of the reported result

| **Signalling questions** | **Comments** | **Response options** |
| --- | --- | --- |
| **5.1 Were the data that produced this result analysed in accordance with a pre-specified analysis plan that was finalized before unblinded outcome data were available for analysis?** | There is no reference to a registered protocol. | PN |
| **Is the numerical result being assessed likely to have been selected, on the basis of the results, from...** |  |  |
| **5.2. ... multiple eligible outcome measurements (e.g. scales, definitions, time points) within the outcome domain?** | No | N |
| **5.3 ... multiple eligible analyses of the data?** | No | N |
| **Risk-of-bias judgement** | **Some concerns** | **Some concerns** |

Overall risk of bias

| **Risk-of-bias judgement** | **High risk of bias** | **High** |
| --- | --- | --- |

| **Study details**   \| **Reference** \| **Preoperative intravenous iron to treat anaemia before major abdominal surgery (PREVENTT): a randomised, double-blind, controlled trial**  *T. Richards, R. R. Baikady, Ben Clevenger, A. Butcher, S. Abeysiri, M. Chau, I. Macdougall, G. Murphy, R. Swinson, T. Collier, L. Van Dyck, J. Browne, A. Bradbury, M. Dodd, R. Evans, D. Brealey, S. Anker, A. Klein* \| \| --- \| --- \|   **Study design**   \| X \| Individually-randomized parallel-group trial \| \| --- \| --- \| \| □ \| Cluster-randomized parallel-group trial \| \| □ \| Individually randomized cross-over (or other matched) trial \|   **For the purposes of this assessment, the interventions being compared are defined as**   \| Experimental: \| 10-42 days before surgery: Intravenous iron was administered as a single 1000 mg dose of ferric carboxymaltose (Ferinject, Vifor Pharma Management, Zurich, Switzerland) in 100 mL normal saline, given as an infusion over 15 min. \| Comparator: \| 100 mL normal saline given as an infusion over 15 min. \| \| --- \| --- \| --- \| --- \|  \| **Specify which outcome is being assessed for risk of bias** \| Incidence of blood transfusion. \| \| --- \| --- \|  \| **Specify the numerical result being assessed.** In case of multiple alternative analyses being presented, specify the numeric result (e.g. RR = 1.52 (95% CI 0.83 to 2.77) and/or a reference (e.g. to a table, figure or paragraph) that uniquely defines the result being assessed. \| Proportion of patients who received perioperative blood transfusion. \| \| --- \| --- \|   **Is the review team’s aim for this result…?**   \| X \| to assess the effect of *assignment to intervention* (the ‘intention-to-treat’ effect) \| \| --- \| --- \| \|  \| to assess the effect of *adhering to intervention* (the ‘per-protocol’ effect) \|   **If the aim is to assess the effect of *adhering to intervention***, select the deviations from intended intervention that should be addressed (at least one must be checked):  □ occurrence of non-protocol interventions  □ failures in implementing the intervention that could have affected the outcome  □ non-adherence to their assigned intervention by trial participants  **Which of the following sources were obtained to help inform the risk-of-bias assessment? (tick as many as apply)**  X Journal article(s) with results of the trial  X Trial protocol  □ Statistical analysis plan (SAP)  □ Non-commercial trial registry record (e.g. ClinicalTrials.gov record)  □ Company-owned trial registry record (e.g. GSK Clinical Study Register record)  □ “Grey literature” (e.g. unpublished thesis)  □ Conference abstract(s) about the trial  □ Regulatory document (e.g. Clinical Study Report, Drug Approval Package)  □ Research ethics application  □ Grant database summary (e.g. NIH RePORTER or Research Councils UK Gateway to Research)  □ Personal communication with trialist  □ Personal communication with the sponsor |
| --- | --- | --- | --- | --- | --- | --- | --- | --- | --- | --- | --- | --- | --- | --- | --- | --- | --- | --- | --- | --- |

## Risk of bias assessment

Responses underlined in green are potential markers for low risk of bias, and responses in red are potential markers for a risk of bias. Where questions relate only to sign posts to other questions, no formatting is used.

**Domain 1: Risk of bias arising from the randomization process**

| **Signalling questions** | **Comments** | **Response options** |
| --- | --- | --- |
| **1.1 Was the allocation sequence random?** | « Randomisation was done by trained staff members using a secure web-based service through the Clinical Trials Unit at the London School of Hygiene & Tropical Medicine. The web-based service was provided by an independent research support organisation. » | Y |
| **1.2 Was the allocation sequence concealed until participants were enrolled and assigned to interventions?** | Yes | Y |
| **1.3 Did baseline differences between intervention groups suggest a problem with the randomization process?** | Characteristics of patients look balanced between the two groups in Table 1, but no p-value is reported. | PN |
| **Risk-of-bias judgement** | **Low risk of bias** | **Low** |

Domain 2: Risk of bias due to deviations from the intended interventions (*effect of assignment to intervention*)

| **Signalling questions** | **Comments** | **Response options** |
| --- | --- | --- |
| **2.1. Were participants aware of their assigned intervention during the trial?** | « To ensure blinding of the participants, their skin was swabbed with iodine, and the study treatment was shielded from vision (light protection bags) and infused through black tubing. » | N |
| **2.2. Were carers and people delivering the interventions aware of participants' assigned intervention during the trial?** | “Unblinded study personnel were responsible for the preparation and administration of the study drug but had no other involvement in the trial.”  « Other clinical and research staff were blinded to the treatment allocated.” | PN |
| **2.3. If Y/PY/NI to 2.1 or 2.2: Were there deviations from the intended intervention that arose because of the trial context?** | N/a | N/a |
| **2.4 If Y/PY to 2.3: Were these deviations likely to have affected the outcome?** | N/a | N/a |
| **2.5. If Y/PY/NI to 2.4: Were these deviations from intended intervention balanced between groups?** | N/a | N |
| **2.6 Was an appropriate analysis used to estimate the effect of assignment to intervention?** | “The primary analysis was by intention to treat, including all randomly assigned patients with data available for the primary endpoints; safety analysis included all randomly assigned patients according to the treatment received.” | Y |
| **2.7 If N/PN/NI to 2.6: Was there potential for a substantial impact (on the result) of the failure to analyse participants in the group to which they were randomized?** | N/a | N/a |
| **Risk-of-bias judgement** | **Low risk of bias** | **Low** |

Domain 3: Missing outcome data

| **Signalling questions** | **Comments** | **Response options** |
| --- | --- | --- |
| **3.1 Were data for this outcome available for all, or nearly all, participants randomized?** | The investigators reported 2 patients in the placebo group, and 1 patient in the intervention group, with missing outcome data for the co-primary endpoint, which includes peri-operative blood transfusion. | Y |
| **3.2 If N/PN/NI to 3.1: Is there evidence that the result was not biased by missing outcome data?** | N/a | N/a |
| **3.3 If N/PN to 3.2: Could missingness in the outcome depend on its true value?** | N/a | N/a |
| **3.4 If Y/PY/NI to 3.3: Is it likely that missingness in the outcome depended on its true value?** | N/a | N/a |
| **Risk-of-bias judgement** | **Low risk of bias** | **Low** |

Domain 4: Risk of bias in measurement of the outcome

| **Signalling questions** | **Comments** | **Response options** |
| --- | --- | --- |
| **4.1 Was the method of measuring the outcome inappropriate?** | No. The outcome assessed in the present meta-analysis is perioperative blood transfusion, and the methods to assess it was appropriate. | N |
| **4.2 Could measurement or ascertainment of the outcome have differed between intervention groups?** | No | N |
| **4.3 If N/PN/NI to 4.1 and 4.2: Were outcome assessors aware of the intervention received by study participants?** | “Other clinical and research staff were blinded to the treatment allocated.” | N |
| **4.4 If Y/PY/NI to 4.3: Could assessment of the outcome have been influenced by knowledge of intervention received?** | N/a | N/a |
| **4.5 If Y/PY/NI to 4.4: Is it likely that assessment of the outcome was influenced by knowledge of intervention received?** | N/a | N/a |
| **Risk-of-bias judgement** | **Low risk of bias** | **Low** |

Domain 5: Risk of bias in selection of the reported result

| **Signalling questions** | **Comments** | **Response options** |
| --- | --- | --- |
| **5.1 Were the data that produced this result analysed in accordance with a pre-specified analysis plan that was finalized before unblinded outcome data were available for analysis?** | « The original study protocol is available online and methodological details of the trial are presented in the appendix and described in brief here. The trial was approved by the UK National Research Ethics Committee, East of England.” | Y |
| **Is the numerical result being assessed likely to have been selected, on the basis of the results, from...** |  |  |
| **5.2. ... multiple eligible outcome measurements (e.g. scales, definitions, time points) within the outcome domain?** | No | N |
| **5.3 ... multiple eligible analyses of the data?** | No | N |
| **Risk-of-bias judgement** | **Low risk of bias** | **Low** |

Overall risk of bias

| **Risk-of-bias judgement** | **Low risk of bias** | **Low** |
| --- | --- | --- |

**Table S4. Risk of bias of included studies**
